# Retraction Note: Long non-coding RNA H19 deteriorates hypoxic-ischemic brain damage by interacting with microRNA-140-5p and STAT3

**DOI:** 10.1186/s11671-023-03830-8

**Published:** 2023-03-20

**Authors:** Qian Lu, Hai Man Hou, Shuo Li, Jing Yuan, Han Liu, Yuming Xu

**Affiliations:** Department of Neurology, The First Affiliated Hospital of Zhengzhou University, 1 Jianshe East Road, Erqi District, Zhengzhou, 450000 Henan China

**Retraction note: Nanoscale Research Letters (2022) 17:43** 10.1186/s11671-022-03666-8

The Editors in Chief have retracted this article due to an overlap with a previously publisher article [1] by different authors and concerns regarding the appearance of western blots. The authors did not respond to requests for further clarification and did not supply raw data or the ethics permit. The Editors, therefore, have lost confidence in the integrity of the article's findings. The authors did not respond to any correspondence from the Editors about this retraction.
